# LRRC8/VRAC channels exhibit a noncanonical permeability to glutathione, which modulates epithelial-to-mesenchymal transition (EMT)

**DOI:** 10.1038/s41419-019-2167-z

**Published:** 2019-12-05

**Authors:** Jonas Friard, Alain Corinus, Marc Cougnon, Michel Tauc, Didier F. Pisani, Christophe Duranton, Isabelle Rubera

**Affiliations:** Université Côte d’Azur, CNRS, Laboratoire de Physiomédecine Moléculaire, LP2M, Labex ICST, Nice, France

**Keywords:** Chloride channels, Ion channel signalling

## Abstract

Volume-regulated anion channels (VRAC) are chloride channels activated in response to osmotic stress to regulate cellular volume and also participate in other cellular processes, including cell division and cell death. Recently, members of the LRRC8 family have been identified as the main contributors of VRAC conductance. LRRC8/VRAC is permeable to chloride ions but also exhibits significant permeability to various substrates that vary strongly in charge and size. In this study, we explored the intriguing ability of LRRC8/VRAC to transport glutathione (GSH), the major cellular reactive oxygen species (ROS) scavenger, and its involvement in epithelial-to-mesenchymal transition (EMT), a cellular process in which cellular oxidative status is a crucial step. First, in HEK293-WT cells, we showed that a hypotonic condition induced LRRC8/VRAC-dependent GSH conductance (P_GSH_/P_Cl_ of ~0.1) and a marked decrease in intracellular GSH content. GSH currents and GSH intracellular decrease were both inhibited by DCPIB, an inhibitor of LRRC8/VRAC, and were not observed in HEK293-LRRC8A KO cells. Then, we induced EMT by exposing renal proximal tubule epithelial cells to the pleiotropic growth factor TGFβ1, and we measured the contribution of LRRC8/VRAC in this process by measuring (i) EMT marker expression (assessed both at the gene and protein levels), (ii) cell morphology and (iii) the increase in migration ability. Interestingly, pharmacologic targeting of LRRC8/VRAC (DCPIB) or RNA interference-mediated inhibition (LRRC8A siRNA) attenuated the TGFβ1-induced EMT response by controlling GSH and ROS levels. Interestingly, TGFβ1 exposure triggered DCPIB-sensitive chloride conductance. These results suggest that LRRC8/VRAC, due to its native permeability to GSH and thus its ability to modulate ROS levels, plays a critical role in EMT and might contribute to other physiological and pathophysiological processes associated with oxidative stress.

## Introduction

Volume-regulated anion channels (VRACs) are ubiquitously expressed proteins that are known to be activated during cell swelling^1^. In hypotonic conditions, VRAC activation leads to an efflux of chloride and organic osmolytes that secondarily drives the regulatory volume decrease (RVD) mechanism. In the absence of osmotic challenge, VRACs are also involved in different physiological processes, including cell proliferation, cell migration and apoptosis. The molecular identity of the membrane proteins generating VRAC conductance has remained unknown until recently. In 2014, two independent studies demonstrated that LRRC8A (leucine-rich repeat–containing) is essential to generate VRAC currents triggered by a hypotonic challenge^2,3^. However, Voss et al. showed that LRRC8A is not sufficient and that other LRRC8 subunits (LRRC8B-E) are necessary to form a constitutive channel^3^. Therefore, VRACs are formed by hetero-hexamers of the different LRRC8 subunits in which LRRC8A is essential^3–6^.

VRACs, similar to their homologous proteins pannexins, are poorly selective channels with a large pore that are permeable to diverse differently charged substrates varying strongly in size. LRRC8/VRACs are permeable to uncharged taurine and negatively charged aspartate and glutamate^2,3^. More recently, LRRC8D was shown to be involved in the transport of small molecules such as the antibiotic blasticidin S^7^, the chemotherapeutic agents cisplatin and carboplatin^8^ and the neurotransmitter GABA^6^. However, the identification, characterization and contribution of chloride channels in physiological or pathophysiological processes have long been limited by the absence of potent selective inhibitors. Currently, the inhibitor DCPIB identified in 2002^9^ was shown to be one of the most powerful molecules to target LRRC8/VRAC conductance^10^.

Plasma membrane transport of the anionic tripeptide glutathione (GSH), the primary cell reducing agent, is one of the mechanisms maintaining intracellular oxidative homeostasis. Few proteins have been identified as GSH transporters, such as members of the ATP binding cassette transporter family, ABCC1-5^11^ and ABCG2^12^, the organic anion transporting polypeptide OATP1-2^13^ and ABCC7/CFTR^14^. Connexin hemichannels are also permeable to GSH^15,16^, promoting regulation of cell junctions and intracellular oxidative status^17^. GSH release during hypo-osmotic stress has already been described in hepatocytes^18^ and in isolated rat thymocytes (Sabirov et al.^19^) and shown to involve DCPIB-sensitive VRAC channels^15,19^.

To explore the role of the putative GSH conductance of LRRC8/VRAC in pathophysiological processes, we took advantage of the well-studied mechanism called epithelial-mesenchymal transition (EMT), in which the generation of oxidative stress is a crucial step. EMT plays crucial roles in embryonic development and tumorigenic processes and is also involved in wound repair or organ fibrosis^20^. EMT is a multistep process characterized by the loss of an epithelial phenotype (polarity and cell-cell junctions) and by the gain of a mesenchymal cell type organization, including (i) cytoskeleton reorganization with de novo expression of α–smooth-muscle actin and vimentin, (ii) synthesis of extracellular matrix proteins such as fibronectin and collagen, which favours tissue stiffness, and (iii) expression of metalloproteases for basement membrane degradation, which results in the acquisition of migratory and invasive phenotypes. The pleiotropic transforming growth factor β1 (TGF-β1) is a key inducer of EMT and is known to induce reactive oxygen species (ROS) production and GSH depletion in many cell types^20^. In renal tubular epithelial cells, the use of antioxidants prevented TGF-β1-induced EMT^21^, demonstrating that the ROS pathway is a signalling mechanism mediating TGF-β1-induced EMT in this cell type.

In this study, we first evaluated the ability of LRRC8/VRAC to transport GSH under hypotonic conditions and then assessed the role of LRRC8/VRAC-mediated GSH efflux in the control of ROS production during the TGF-β1-induced EMT process under isotonic conditions.

## Materials and methods

### Cell culture treatments and inhibitors

We used the immortalized HEK-293 wild-type and HEK-293 LRRC8A-KO cell lines (kindly provided by Pr. TJ Jentsch, Berlin, Germany) (Voss et al.^3^). Cells were classically cultured in DMEM medium culture containing 10% serum and penicillin/streptomycin (50 U/ml). Cultures were maintained in a water-saturated atmosphere of 5% CO_2_/95% air at 37 °C before use. Cells were used between passage 15 and 25.

EMT experiments were performed using HK-2 cells, a human proximal tubule epithelial cell line (a gift from Dr. Eric Rondeau, Paris). Cells were cultured in DMEM-F12 culture medium supplemented with 10% foetal bovine serum (FBS), 100 U/ml penicillin and 100 µg/ml streptomycin. Cultures were classically maintained in a water-saturated atmosphere of 5% CO2/95% air. At 80% confluency, cells were incubated in basal medium in the absence of FBS for 24 h. Cells were then incubated for 24 h with TGF-β1 (2.5 ng/ml; R&D Systems). Cells were used between passage 10 and 20.

DCPIB, 4-(2-butyl-6,7-dichloro-2-cyclopentyl-indan-1-on-5-yl) oxobutyric acid (Sigma-Aldrich), was used at final concentrations of 10–20 µM (stock solution 10 mM in DMSO); NPPB, 5-nitro-2-(3-phenylpropylamino) benzoic acid (Sigma-Aldrich), was used at a final concentration of 100 µM.

Gene silencing of LRRC8A was performed using small interfering LRRC8A (SMARTpool: ON-TARGETplus human siRNA LRRC8A; Dharmacon) or siRNA negative control (medium GC duplex, Invitrogen). Cells were transfected in HK-2 cells using Lipofectamine 3000 (Invitrogen) according to the manufacturer’s protocol 96 h before experimentation.

### Kinetics of intracellular GSH variation

CMFDA (diacetate of 5-chloromethylfluoresceine; Thermo Fisher Scientific) is a green fluorescent probe sensitive to reduction. As GSH is the main intracellular reducing agent, variation of CMFDA fluorescence is an indirect indicator of intracellular GSH levels. At confluency, the culture media was removed before the experiment and replaced by HBSS. The experiments were performed in HBSS medium. Cells were loaded with 1 µM CMFDA for 30 min before the experiment. Basal fluorescence was recorded for 10 min, and the cells were challenged by hypotonic media (100 mOsm l^−1^) for 50 min. The fluorescence was measured every 1.5 min at 520 nm (excitation 490 nm) using a Synergy HT Automated Microplate Reader (Biotek).

### Quantification of intracellular GSH content

The intracellular GSH cell contents were quantified using a GSH/GSSG kit (Biovision, Mountain View, CA, USA), a fluorescent quantification method. At confluency, the culture medium was removed before the experiment and replaced by HBSS. The experiments were performed in HBSS medium. Briefly, cells cultivated in 60 mm dishes were homogenized in an ice-cold GSH buffer, and proteins were removed by precipitation using perchloric acid and centrifugation. After neutralization of the supernatants using KOH, the GSH contents were determined using the O-phthalaldehyde (OPA) fluorescent probe. In the assay, OPA reacts with GSH (not GSSG), generating fluorescence proportional to the GSH content. The fluorescence was measured at 420 nm (excitation 340 nm) using a Synergy HT Automated Microplate Reader. The exact protein content in each well was measured using Bio-Rad protein assays.

### Patch-clamp recordings

The ruptured whole-cell configuration of the patch-clamp technique was used to measure chloride and GSH conductances. Cell currents from isolated cells were recorded using an EPC 10 amplifier (HEKA Elektronik, Lambrecht (Pfalz), Germany). Cells were held at −50 mV, and 400 ms pulses from −100 to +100 mV were applied in 20 mV increments. I/V relationships are expressed as the mean current amplitudes measured at all potentials at 6–10 ms after the pulse onset. The offset potentials between both electrodes were zeroed before sealing and corrected for liquid junction potentials as previously described^22^.

For chloride current recordings, the pipette solution contained (in mM) 140 NMDGCl, 10 HEPES (pH 7.4), 5 EGTA and 5 MgATP (290 mOsm l^−1^). For GSH current recordings, the pipette solution contained (in mM) 140 NMDG-GSH, 10 HEPES (pH 7.4), 5 EGTA and 5 MgATP (290 mOsm l^−1^). For GSH current recordings and to avoid any contamination of the pipette solution with chloride ions, a 140 NMDG-GSH solution was prepared using 140 mM of NMDG, 10 mM of HEPES, 5 mM of MgATP and 5 mM of EGTA that was adjusted to pH 7.2 by addition of GSH (290 mOsm l^−1^).

The control NMDGCl bath solution contained (in mM) 140 NMDGCl, 10 HEPES (pH 7.4, HCl), 1 CaCl_2_, 1 MgCl_2_, and ~70 mannitol (340 mOsm l^−1^). This solution was designed to prevent spontaneous activation of VRAC currents. Hypo-osmotic NMDGCl solution (270 mOsm l^−1^) was obtained using the same solution without the addition of mannitol. The osmolarity of all the patch-clamp solutions was tested and adjusted if necessary using an osmometer (automatic type 13 Roebling). GSH permeability (P_GSH_/P_Cl_) was calculated with the Goldman-Hodgkin-Katz equation after correction of the liquid junction potentials and taking into consideration that the mobility for GSH was 0.24^19^.

### Real-time PCR analysis

Total RNA was extracted from cells using the NucleoSpin® RNA kit (Macherey-Nagel, Germany) following the manufacturer’s protocol. Reverse transcription was performed using 1.5 µg of each RNA sample, M-MLV-RT (Promega) and a mixture of Random Primers (Promega) and Oligo(dT)15 Primer (Promega). For real-time PCR analysis, experiments were performed using 2× SensiFAST SYBR HI-ROX mix (Bioline) on a Step One Plus Real-Time PCR System (Applied Biosystems). The specificity of the amplification reactions was confirmed by melting curve analysis. The expression levels of selected genes were calculated using the comparative Ct (ΔΔCt) method and normalized by the expression of the housekeeping gene 36B4. qPCR primers are available upon request to the author.

### Intracellular ROS measurements

The level of cellular oxidative stress was measured using the fluorescent probe (5-and-6)-carboxy-2′,7′-dichlorodihydrofluorescein diacetate (carboxy-H2DCFDA). Briefly, confluent HK-2 cells were incubated at 37 °C for 30 min in the presence of carboxy-H2DCFDA (50 μM) and gently washed in serum-free culture medium. Cells were then incubated in the absence or presence of either TGF-β1 or DCPIB in HBSS media. Variations in fluorescence were measured every 2 min at 538 nm using a Synergy HT Automated Microplate Reader.

### Western blot analysis

For Western blotting, equivalent amounts of protein per lane (20 µg) were separated by SDS-PAGE on 10% acrylamide gels. Thereafter, the protein was transferred to a PVDF membrane (Millipore, Billerica, MA). The membranes were probed with primary anti-N-Cadherin (dilution 1:1000; BD Biosciences, 610921) and mouse anti-ß-actin antibodies (dilution 1:10,000, Sigma, A1978) and then probed with horseradish peroxidase-conjugated secondary antibody (Perkin Elmer, dilution 1:4000, NEF822001EA).

### Immunofluorescence labelling

Cells were grown on sterile dishes with a cover glass bottom (Fluorodish) and serum-starved for 24 hours prior to treatment with 2.5 ng/ml TGF-β1 for 24 h. Cells were then fixed with 4% paraformaldehyde for 10 min at room temperature and permeabilised with 0.1% Triton X-100 for 10 min. After blocking with 3% BSA for 1 h, cells were incubated with the primary antibody against N-Cadherin (BD Biosciences, 1:100 dilution) or vimentin (Cell Signaling, 1:100 dilution) overnight at 4 °C. Cells were then incubated with Alexa Fluor® 488 conjugated goat anti-mouse or anti-rabbit secondary antibody at a 1:1000 dilution for 1 h at room temperature in the dark. To ensure the specificity of our results, negative controls with no primary antibody or no secondary antibody were included. For nuclear counterstaining, cells were incubated with Hoechst 33342 (1:10,000 dilution) for 15 min. Cells were visualised with a Carl Zeiss Axiover D1 inverted microscope using a 40× LD Plan-Neofluar objective.

### Morphological analysis

Morphological analysis was performed using the shape descriptors of ImageJ software on images taken with a Carl Zeiss Axiover D1 inverted microscope with a 20× objective. Circularity was calculated by 4*π* × ([Area])/([Perimeter]^2^) and the aspect ratio by ([Major Axis])/([Minor Axis]) for individual cells.

### Wound healing assay

Confluent HK-2 cells were growth-arrested in serum-free medium for 24 h, and then, the monolayer was linearly scratched with a sterile 200-μl pipette tip, washed twice with 1x PBS to remove nonadherent cells, and treated as indicated. Wound closure was monitored by phase contrast microscopy. Phase-contrast images of wound closure were recorded at 0 and 24 h after scratching. Wound closure was quantified using ImageJ software at 24 h after wounding. Values are expressed as the ratio of the initial wound area. The average wound area relative to the initial wounding (0 h) was determined in three independent triplicate assays.

### Statistical analysis

Graphics and data analysis have been performed with GraphPad Prism 6 (GraphPad Software). Sample size was chosen based on the need for statistical power. Quantitative data are expressed as box plots. For patch-clamp analysis, comparison between two groups has been made using Mann–Whitney and Wilcoxon paired tests. ANOVA with Bonferroni’s multiple comparison post hoc test has been performed for morphological analysis. For Western blot analysis, the data were compared, before normalization to the control condition, with the Friedman paired test with Dunn’s multiple comparison post hoc test. For others group analysis, Kruskal-Wallis tests with Dunn’s multiple comparison post hoc test have been used. Symbols for *P*-values are given in each figure legend.

## Results

### Hypotonic shock induced GSH depletion in LRRC8A-expressing cells

**Fig. 1:**
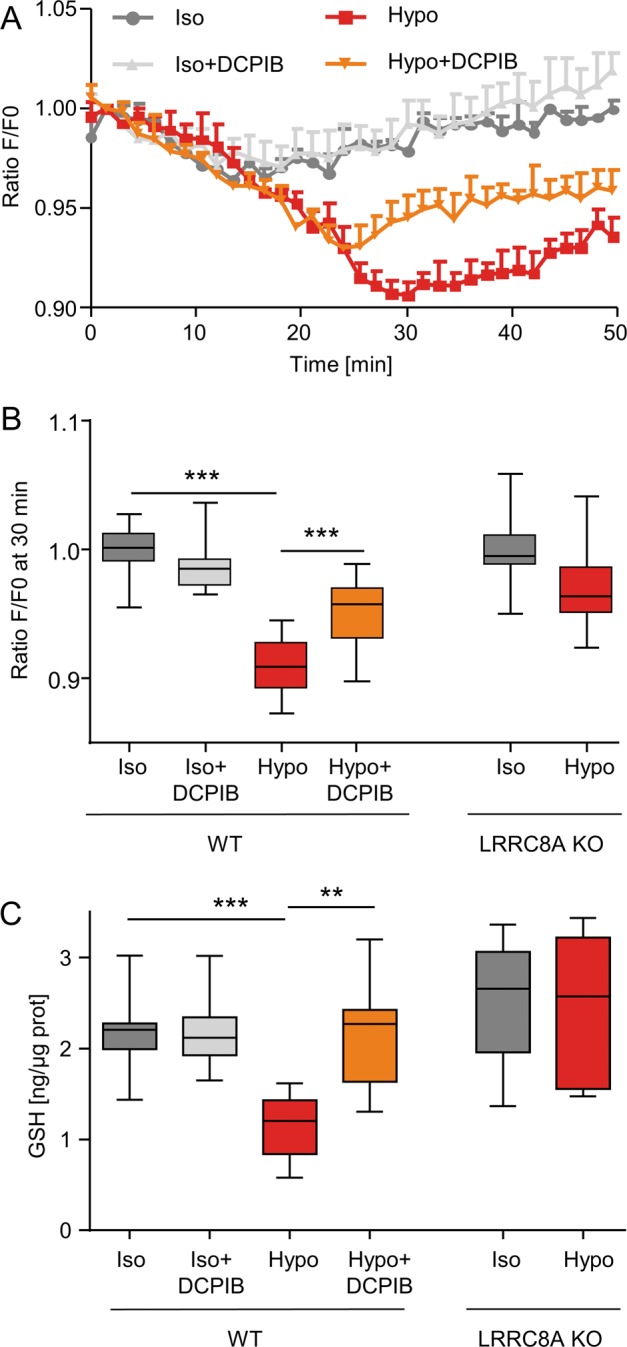
Variations in GSH intracellular contents in WT and LRRC8A-KO HEK293 cells during hypotonic shock. **a** Kinetics of fluorescence variations reflecting the intracellular GSH variations measured in WT HEK293 cells exposed to isotonic (300 mOsm l^−1^) and hypotonic (100 mOsm l^−1^) solutions in the absence or presence of DCPIB (20 µM). Cells were loaded for 30 min in the presence of the fluorescent GSH probe (CMFDA). After an equilibrium period of 10 min, fluorescence variations of the confluent monolayers were monitored every 90 s for a total period of 50 min. **b** Normalized fluorescent variations measured in WT and LRRC8A KO HEK293 cells under iso-osmotic or hypotonic solutions in the absence or presence of DCPIB. Box plots display values taken at a fixed time of 30 min after the beginning of the hypotonic exposure (*n* = 18 measurements from six independent experiments for WT and *n* = 11 measurements from four independent experiments for LRRC8A KO HEK293 cells, Kruskal-Wallis with Dunn’s multiple comparison post hoc test, ***p < 0.001). **c** Effect of hypotonic exposure (30 min, 100 mOsm l^−1^) on the intracellular GSH content measured in WT and LRRC8-KO HEK293 cells using the fluorescent probe o-phthalaldehyde (OPA). Confluent monolayers were incubated in isotonic (300 mOsm l^−1^) and hypotonic (100 mOsm l^−1^) solutions in the absence or presence of DCPIB (20 µM). Box plots represent intracellular GSH content obtained from 12–15 measurements (four independent experiments) for WT HEK293 and nine measurements (three independent experiments) for LRRC8A KO HEK293 cells. Kruskal-Wallis with Dunn’s multiple comparison post hoc test was used with **p < 0.01, ***p < 0.001.

LRRC8/VRAC activation is known to be triggered by hypotonic cell swelling. Our first aim was to measure the variation of intracellular GSH content during hypotonic shock using an intracellular fluorescent GSH probe (CMFDA) in HEK293 cells. In these cells, hypotonic solution induced a significant decrease in fluorescence, which was maximal at 30 min, suggesting a decrease in intracellular GSH content (Fig. 1a). Interestingly, DCPIB (20 µM), a well-known inhibitor of LRRC8/VRAC, partially blocked this decrease (Fig. 1a, b). Then we analyzed hypotonic-induced fluorescence variations measured at 30 min in WT and LRRC8A-KO HEK293 cells. Hypotonic solution exposure induced a significant decrease in fluorescence only in WT cells (Fig. 1b).

Since these measurements using the CMFDA fluorescent probe are not quantitative, we measured the intracellular GSH content using direct extraction and quantification with the OPA probe. Figure 1c illustrates the results obtained in WT and LRRC8A-KO cells. Hypotonic shock induced a significant decrease of ~35% of the intracellular GSH content in WT cells and had no effect in LRRC8A-KO cells. In WT HEK293 cells, DCPIB (20 µM) has no effect on GSH level under isotonic condition but abolished the hypotonic-induced GSH decrease.

To validate that CMFDA or OPA fluorescence changes are ascribed to changes in GSH, both methods were performed in GSH-depleted cells. WT HEK cells were treated 24 h with buthionine sulfoximine (BSO, 0.5 mM), an inhibitor of GSH synthesis). As illustrated in Fig. S1, BSO induced a dramatic decrease in both CMFDA fluorescence and OPA fluorescence validating both methods to detect intracellular GSH variations.

### Hypotonicity triggered GSH conductance through the LRRC8/VRAC channel

**Fig. 2:**
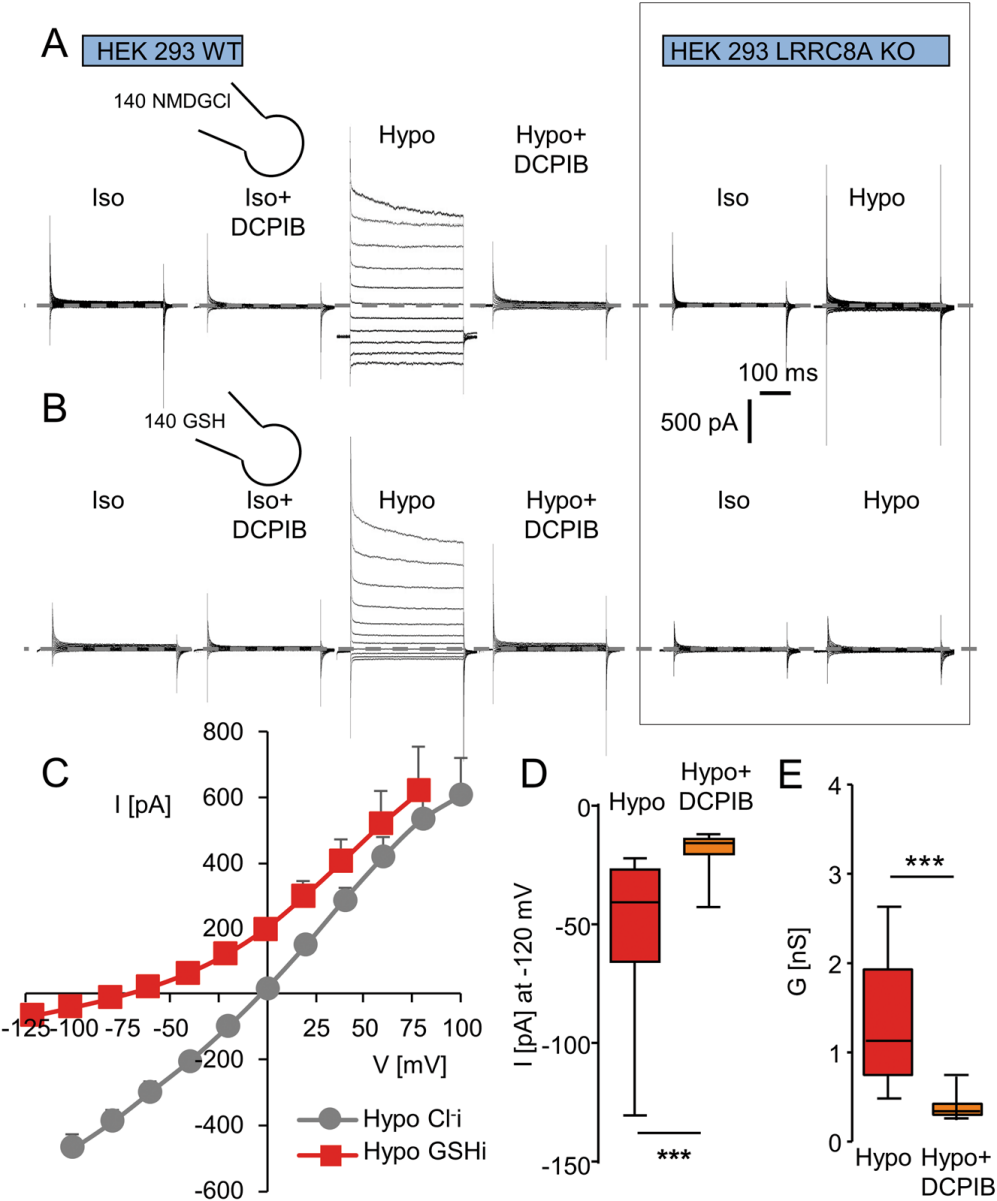
Chloride and GSH permeabilities of the LRRC8/VRAC conductance measured in WT and LRRC8A-KO HEK293 cells. **a** Whole-cell Cl^−^ currents recorded in WT (left) and LRRC8A-KO HEK 293 cells (right), under control conditions (Iso, 340 mOsm l^−1^), in the presence of DCPIB (10 µM, Iso + DCPIB) and after replacing the bath with a hypotonic solution (Hypo, 270 mOsm l^−1^). Once the Cl^−^ conductance was fully developed (3–4 min), DCPIB was perfused. **b** Whole cell currents recorded as in (**a**) after replacement of the NMDGCl pipette solution with a GSH pipette solution. Bath solution compositions are the same as in (**a**) containing NMDG^+^/Cl^−^ as major ions. **c** Mean current/voltage relationships measured in HEK293 WT recorded after the stabilization of the VRAC Cl^−^ current (hypo). Current values were measured 6–10 ms after the onset pulse. The means I/V relationship recorded under asymmetrical pipette and bath solution is also plotted (GSH pipette solution versus Cl bath solution). Note that all the relationships illustrated are corrected by the junction potential calculated for each experimental condition. **d**, **e** Box plots illustrating the remaining GSH current (**d**, measured at −120 mV) and the GSH conductance (**e**, calculated between −100 and −60 mV) and their sensitivity to DCPIB exposure (20 µM) at −120 mV. Currents were recorded with GSH pipette solution and hypo NMDGCl bath solution as in (**b**). Records were obtained from 13 to 14 individual cells (Mann–Whitney and Wilcoxon paired tests were used, ***p < 0.001).

Using a whole-cell approach, we explored the GSH permeability of the LRRC8/VRAC channel in WT versus LRRC8A-KO HEK293 cells (Fig. 2). Figure 2a illustrates whole-cell recordings using NMDGCl pipette solution. In WT cells, decreasing extracellular osmolarity from 340 to 270 mOsm l^−1^ (hypotonic exposure) induced a chloride current in 3–5 min with strong inactivation at positive potentials (Fig. 2c). DCPIB (10 µM, a fully washable VRAC inhibitor), fully inhibited this hypotonic-induced chloride conductance (Fig. 2a). As previously demonstrated^3,10^, LRRC8A-KO cells failed to develop any chloride current when exposed to hypotonic solution. Then, we performed similar experiments by replacing 140 mM NMDGCl in the pipette with 140 mM GSH (Fig. 2b). Hypotonicity induced an outwardly rectifying current with a strong rectification in 3–5 min (E_rev_ after correction for the liquid junction is close to −66.8 mV, *n* = 14, Fig. 2c). At −120 mV, we recorded a small negative current that was sensitive to DCPIB (10 µM, Fig. 2d). Similarly, DCPIB induced an inhibition of the GSH-mediated conductance (Fig. 2e). These results suggest that this small current is mainly supported by GSH efflux. The calculated relative permeability P_GSH_/P_Cl_ was 0.08 ± 0.01 (*n* = 14, Fig. S2b). Interestingly, with GSH in the pipette, exposure to hypotonic solution had no effect in LRRC8A-KO cells (Fig. 2b, no increase in the current at negative or positive potentials).

**Fig. 3:**
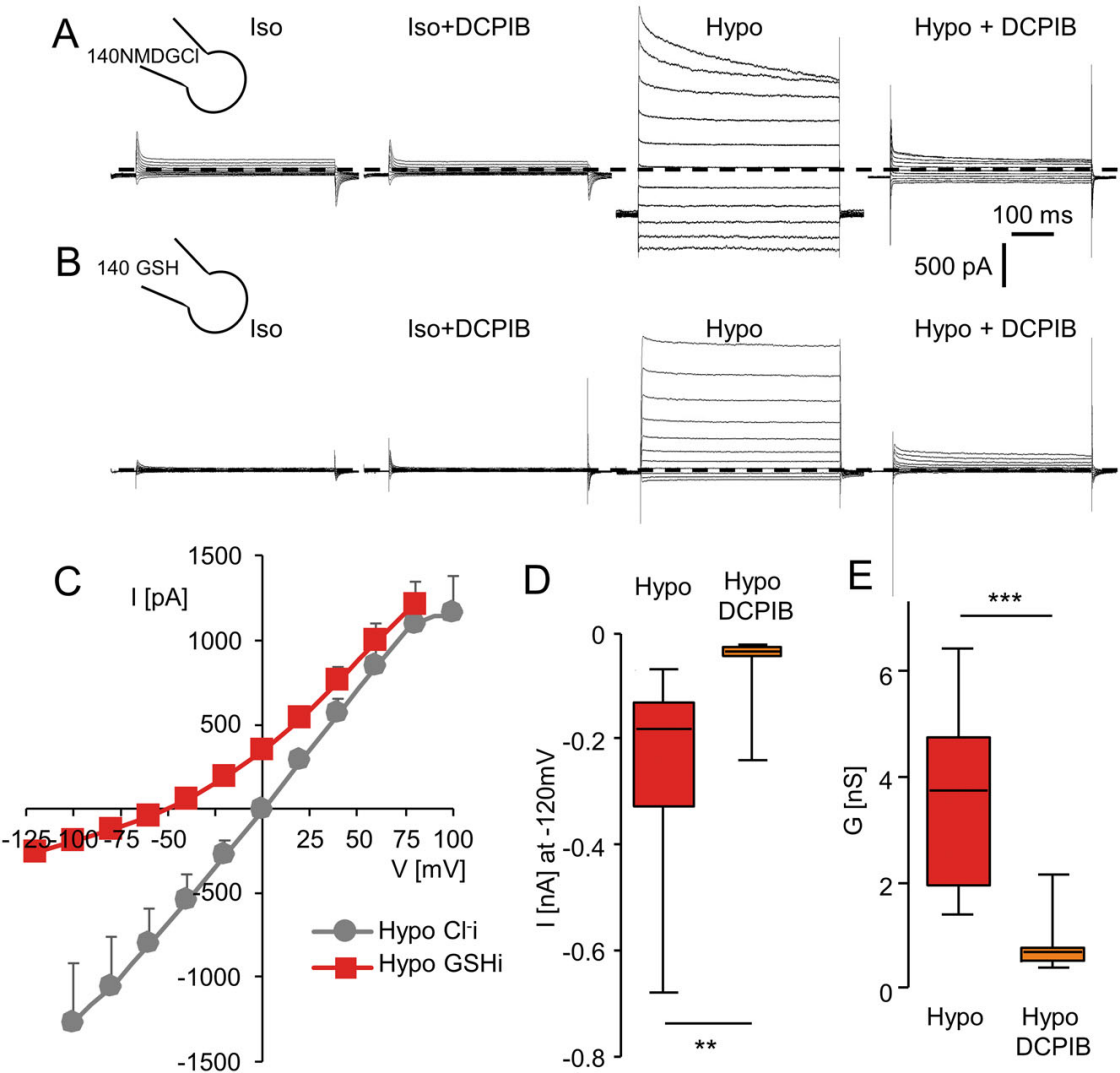
Chloride and GSH permeabilities of the LRRC8/VRAC conductance measured in HK2 kidney cells. **a** Whole-cell Cl^−^ currents recorded in HK2 cells under control conditions (Iso, 340 mOsm l^−1^), in the presence of DCPIB (10 µM, Iso + DCPIB), after replacing the bath with a hypotonic solution (Hypo, 270 mOsm l^−1^) and after the addition of DCPIB (10 µM). Normal bath solution and pipette solution (290 mMosm l^−1^) contained 140 mM of NMDGCl. **b** Whole cell currents recorded as in (**a**) after replacement of the NMDGCl pipette solution with an iso-osmotic GSH pipette solution. Bath solution compositions are the same as in (**a**). **c** Mean I/V relationships measured in HK2 cells recorded in control conditions (Iso) and in hypotonic condition (Hypo) with chloride or GSH pipette solutions. Current values were measured 6 10 ms after the onset pulse. **d**, **e** Box plots illustrating the remaining GSH current (**e**, measured at −120 mV) and the GSH conductance (**d**, calculated between −100 and −60 mV) and their sensitivity to DCPIB exposure (20 µM). Currents were recorded with GSH pipette solution and hypo NMDGCl bath solution as in (**b**). Records were obtained from 8 to 10 individual cells (Mann–Whitney test was used, **p < 0.01).

We then analyzed LRRC8/VRAC conductance in human kidney tubular epithelial cells. HK2 cells mainly expressed mRNA of LRRC8A/D subunits. However, while mRNA expression of LRRC8B, C, D, E subunits are similar in HEK293 and HK2 cell lines, LRRC8A mRNA subunit is ~5 times more expressed in HK2 cells (Fig. S2a). As expected, hypotonic-activated chloride currents were inhibited by DCPIB when recorded with NMDGCl pipette and bath solution (Fig. 3a). When pipette chloride was replaced by GSH, E_rev_ shifted towards negative values (−58.0 mV, *n* = 10, Fig. 3c), and a DCPIB-sensitive current was recorded at negative potentials (Fig. 3d) and the hypotonic-induced GSH conductance was inhibited by DCPIB (Fig. 3e). The calculated relative permeability P_GSH_/P_Cl_ was 0.11 ± 0.02 (*n* = 10, Fig. S2b). During this ion substitution, the outward currents carried by chloride remained unaffected.

Altogether, these results suggest that LRRC8/VRAC is permeable to GSH and can modulate intracellular GSH content.

### TGFβ1 induces VRAC activation, GSH depletion and ROS increase in HK2 cells

**Fig. 4:**
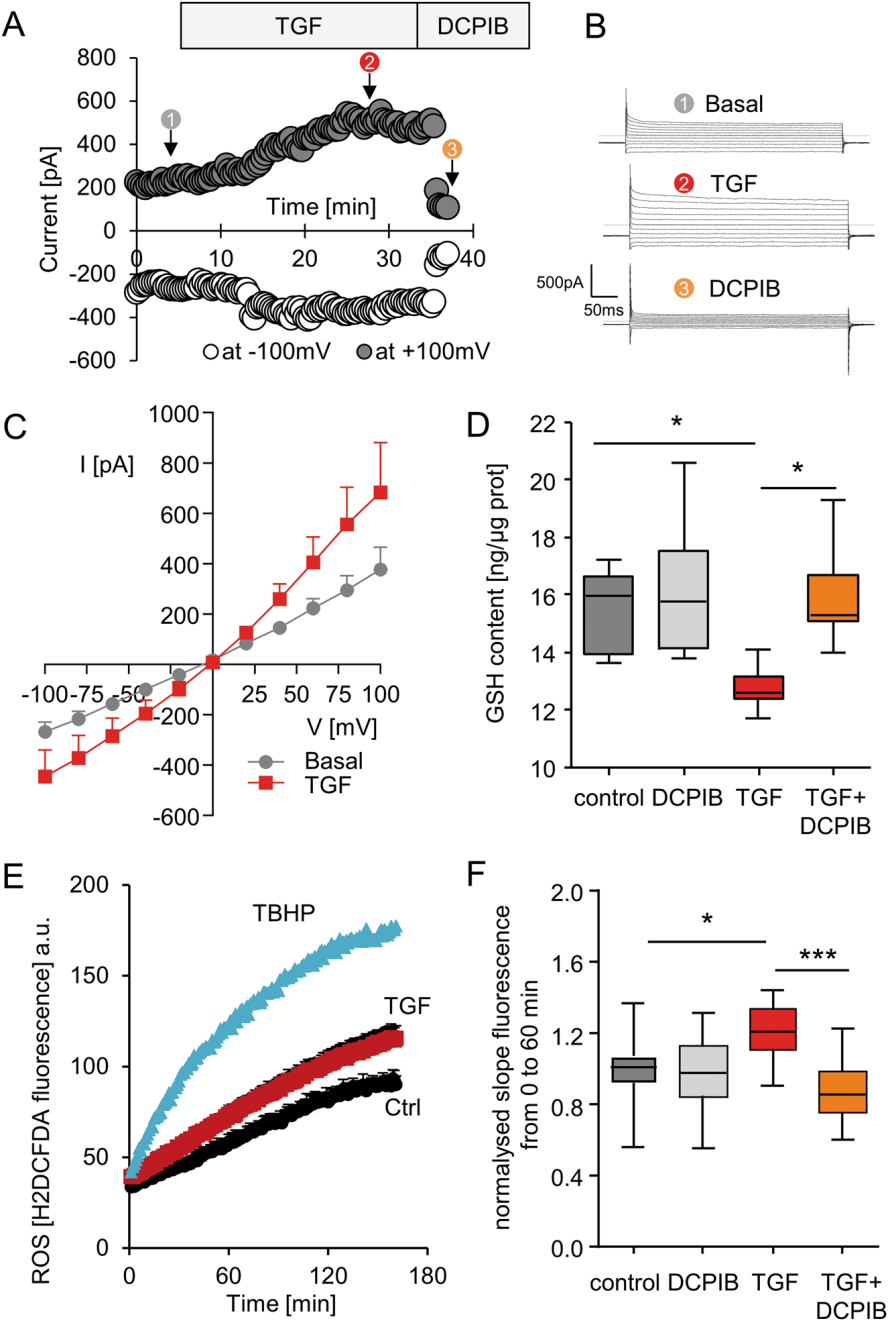
Effect of TGFβ1 on LRRC8/VRAC channel activation and its contribution to the modulation of intracellular GSH content and ROS production. **a** Whole-cell Cl^−^ currents recorded at −100 and +100 mV as a function of TGFβ1 (2.5 ng/ml) exposure time and followed by a subsequent DCPIB treatment (10 µM). **b** Whole cell currents recorded from −100 to +100 mV by step of 20 mV for each time point (identified by 1, 2 and 3 in Fig. 4a). **c** Mean current-voltage (I/V) relationships measured before and after a 30 min TGF β1 exposure. Records were obtained from five individual cells. **d** Box plots illustrating the intracellular GSH concentrations measured in control conditions or after exposure to TGFβ1 (2.5 ng/ml; 4 h) in the absence or presence of DCPIB (10 µM) using the OPA fluorescent probe. Data were obtained from 6 to 7 measurements (three independent experiments), Kruskal-Wallis with Dunn’s multiple comparison post hoc test was used with *p < 0.05. **e** Kinetics of ROS production measured in HK2 cells as a function of time. Cells were incubated for 1 h in the presence of H_2_-DCFDA and washed, and fluorescence was measured every 2 min for 180 min in control conditions (black) or in the presence of TGFβ1 (red, 2.5 ng/ml). A positive control was obtained by adding 1 mM of tBHP as an external oxidant molecule (blue). **f** Box plots illustrating the normalized slope fluorescence (calculated for a period of 60 min after the addition of the various substances) as measured in (**e**), corresponding to cells exposed to DCPIB or TGF alone and to both substances simultaneously (TGF + DCPIB).Values were obtained from 17 to 18 measurements (five independent experiments), Kruskal-Wallis with Dunn’s multiple comparison post hoc test was used with *p < 0.05, ***p < 0.001.

In addition to hypotonic shock, LRRC8/VRAC channels can be activated by other stimuli^23,24^. Because the profibrotic growth factor TGFβ1 has been shown to decrease GSH in various types of cells^20^, we aimed to determine whether TGFβ1 could modulate LRRC8/VRAC activity. We thus recorded the Cl^−^ current triggered by TGFβ1 in iso-osmotic conditions in HK-2 cells. TGFβ1 exposure (2.5 ng/ml) induced a chloride current whose amplitude increased with time and reached a maximum after ~20 min; this current exhibited biophysical characteristics of the LRRC8/VRAC: outward rectification, inactivation at positive potentials and sensitivity to DCPIB (Fig. 4a–c). Interestingly, the VRAC current triggered by TGFβ1 has a slower activation and a current amplitude at +100 mV weaker than that recorded under hypotonic challenge.

We then measured the intracellular levels of GSH and ROS in TGFβ1-stimulated HK-2 cells. TGFβ1 treatment (2.5 ng/ml, 4 h) decreased the intracellular GSH level by 22%, an effect almost completely prevented by coincubation with DCPIB (Fig. 4d). Figure 4e shows the time course of ROS formation measured in HK-2 cells submitted to TGFβ1 treatment. The fluorescence emitted by the ROS-sensitive probe CM-H_2_DCFDA increased soon after the application of TGFβ1 compared to the control condition. A positive condition was generated by exposing the cell to a strong ROS donor (TBHP, tert-butyl hydroperoxide, 1 mM). To precisely compare the variation of the relative fluorescence according to the experimental conditions, we calculated the initial slope variation between *t* = 0 min and *t* = 60 min, and the relative values are presented in Fig. 4f. As a result, acute TGFβ1 induced a significant increase in ROS production. This TGFβ1-induced ROS production is abolished by coincubation with DCPIB. DCPIB alone had no significant effect on ROS production compared to the control.

These results suggest that TGFβ1-induced ROS production might be due to an efflux of reduced GSH mediated by LRRC8/VRAC.

### LRRC8/VRAC inhibition attenuates TGFβ1-induced EMT marker expressions

TGFβ1 is a key inducer of EMT. EMT is characterized by loss of epithelial markers and gain of mesenchymal markers, resulting in the acquisition of migratory and invasive phenotypes. Hence, to test the ability of LRRC8/VRAC to modulate EMT, we assessed the effect of DCPIB on molecular markers as well as functional attributes of EMT. To exclude the toxic effect of DCPIB, experiments were performed to estimate the viability of HK-2 cells after 24 h for increasing concentrations of this drug (1, 3, 10, 20, 30 and 100 µM). Concentrations of DCPIB up to 20 µM had no toxic effects on HK-2 cells (Fig. S3).

**Fig. 5:**
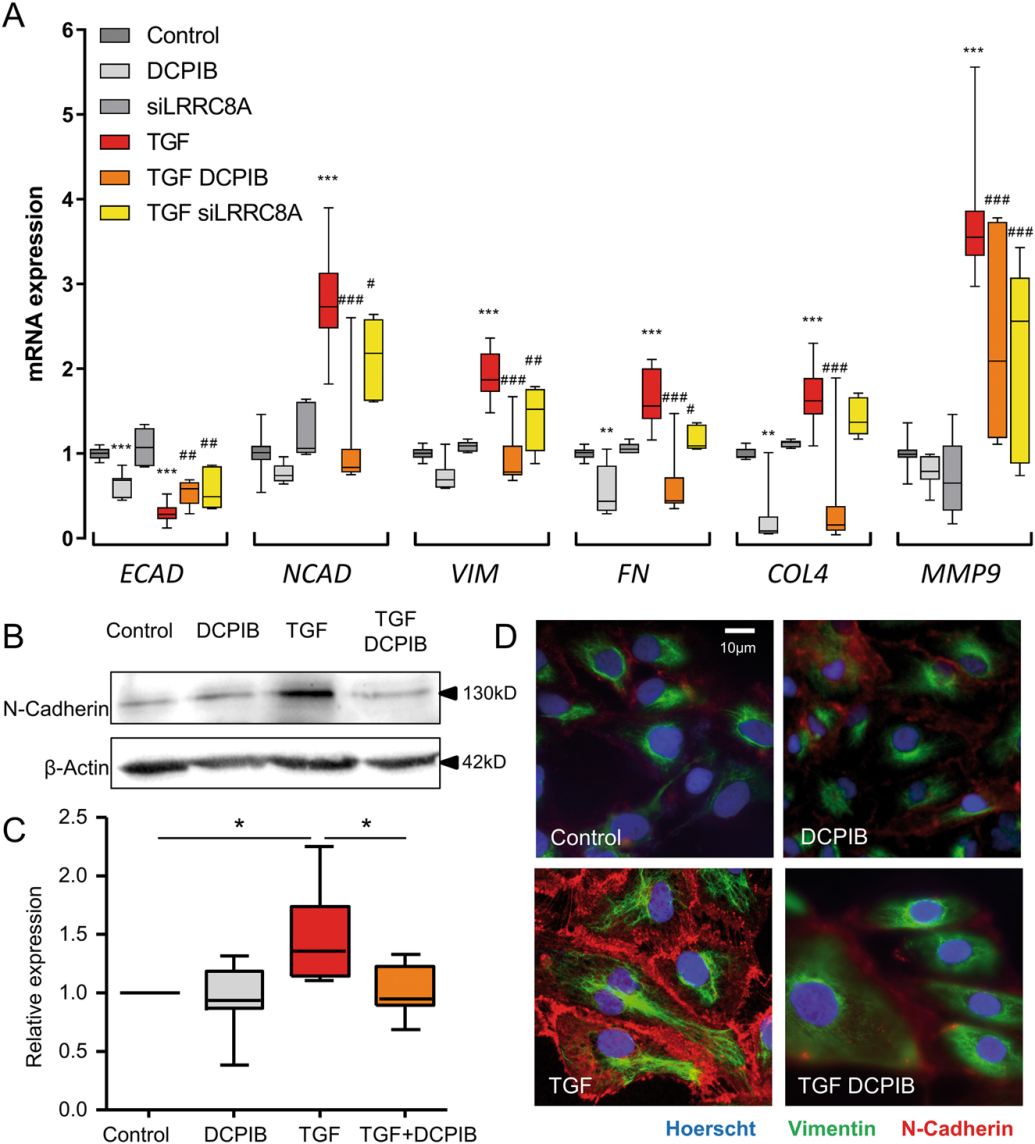
DCPIB or LRRC8A knockdown alters the expression of EMT markers after TGFβ1 treatment in HK-2 cells. **a** Fold increase in mRNA levels of *ECAD* (E-cadherin), *NCAD* (N-cadherin), *VIM* (Vimentin), *FN* (Fibronectin), *COL4* (Collagen IV) and *MMP9* (Matrix Metalloproteinase-9) in HK-2 cells cultured with or without TGFβ1 (2.5 ng/ml) for 24 h in the presence or absence of DCPIB (20 µM) or after silencing of LRRC8A (siRNA). *36B4*-normalized mRNA levels in control cells were used to set the baseline value at unity. Box plots illustrating the mRNA fold increase of 5–13 experiments from five independent cell cultures. Kruskal-Wallis with Dunn’s multiple comparison post hoc test was used with **p < 0.01, ***p < 0.001 vs control; ^#^*p* < 0.05, ^##^*p* < 0.01, ^###^*p* < 0.001 vs TGF. **b**, **c** Protein expression of N-cadherin in cells treated with TGFβ1 (2.5 ng/ml) for 24 h in the presence or absence of DCPIB (20 µM). β-actin was used as a loading control. Representative Western blots (**b**) and corresponding quantitative analysis (**c**) performed on five independent experiments. The results are expressed as the n-fold increase over the control and Friedman + Dunn statistic test was used with *p < 0.05. **d** Immunofluorescence staining of N-cadherin and vimentin proteins. Nuclei were stained with Hoechst 33342 dye. Cells were treated with or without TGFβ1 (2.5 ng/ml) for 24 h in the presence or absence of DCPIB (20 µM) as indicated. Scale bar: 10 µm.

We first investigated whether DCPIB (20 µM) affected TGFβ1-induced EMT by assessing EMT marker gene expression by real-time PCR. TGFβ1 (2.5 ng/ml, 24 h) decreased the transcript level of the epithelial marker E-cadherin (ECAD) and increased the transcript expression of the mesenchymal marker N-cadherin (NCAD), markers of the cytoskeleton (vimentin (VIM)), extracellular matrix structural elements (fibronectin (FN) and collagen IV (COL4)) and metalloproteases (MMP9), consistent with the induction of EMT events (Fig. 5a). However, in DCPIB-treated cells, TGFβ1-induced upregulation of the mRNA levels of these markers and downregulation of ECAD were strongly inhibited (Fig. 5a). DCPIB alone induced a downregulation of the ECAD, FN and COL4 mRNA expression. To confirm a direct contribution of LRRC8/VRAC, we performed RNA interference-mediated inhibition of LRRC8A (siLRRC8A). The efficiency of this silencing was confirmed by a strong decrease of LRRC8A mRNA expression (Fig. S4a) and assessed by whole-cell recordings showing that siLRRC8A-transfected HK-2 cells failed to develop LRRC8/VRAC currents (Fig. S4b–d). Silencing of LRRC8A significantly inhibited the effect of TGFβ1 on EMT mRNA markers (Fig. 5a).

Interestingly, the ROS scavenger NAC (N-acetylcysteine, 5 mM), a precursor of GSH, or direct addition of GSH (15 mM) inhibited TGFβ1-induced downregulation of epithelial markers and upregulation of mesenchymal markers (Fig. S5a, b), confirming the link between GSH levels and EMT events. Moreover, the non-thiol antioxidant α-tocopherol (100 µM) also prevented EMT (Fig. S3c).

Induction of NCAD expression was also measured at the protein level by Western blot analysis. As a result, TGFβ1 exposure significantly increased NCAD protein expression, which is consistent with its mRNA levels (Fig. 5b, c). DCPIB fully abrogated the TGFβ1-induced expression of NCAD (Fig. 5b, c). Furthermore, we performed immunofluorescence staining of NCAD and VIM to confirm the reorganization of the cells that undergo EMT upon TGFβ1 treatment. Indeed, TGFβ1 induced a strong increase in NCAD fluorescent labelling, mainly at the cell’s border, while the cytoskeleton marker vimentin appeared to be organised in fibres. DCPIB exposure abrogated the expression of NCAD and prevented the formation of VIM fibres (Fig. 5d).

### LRRC8/VRAC inhibition attenuates TGFβ1-induced EMT phenotypes

**Fig. 6:**
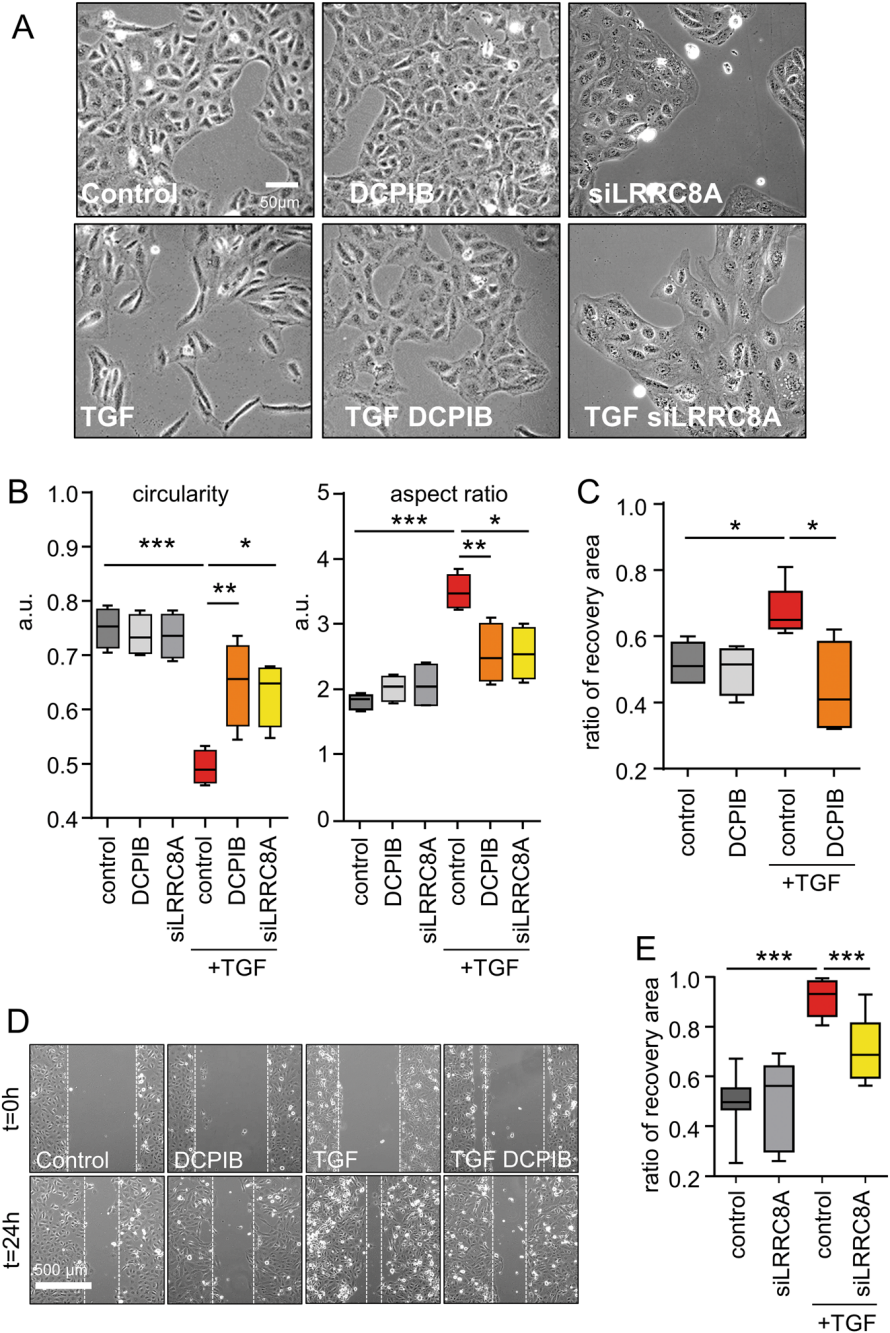
Pharmacological inhibition of VRAC or LRRC8A knockdown attenuates TGF β1-induced cell morphology changes, migration and invasion. **a** Representative micrograph (phase-contrast microscopy) illustrating the morphological changes induced by TGFβ1 treatment (2.5 ng/ml, 24 h) and the inhibitory effects of DCPIB (20 µM) or LRRC8A gene silencing. **b** Box plots illustrating variations in circularity (left) and aspect ratio (right) as two indicators of morphological changes induced by TGFβ1 treatment concomitant with DCPIB exposure or LRRC8A knockdown (siRNA). The two parameters were analyzed with ImageJ shape descriptor software. Data were obtained from 60 cells (four independent experiments). ANOVA with Bonferroni’s multiple comparison post hoc test statistical test was used with *p < 0.05, **p < 0.01. **c**, **d** Box plots (**c**) and micrographs (**d**) illustrating the effect of pharmacological inhibition of LRRC8/VRAC in migration processes using wound healing experiments. HK2 cells were scratched and wounded after a 24 h starving period without serum. Wound closure was monitored using an inverted microscope for 24 h with or without TGFβ1 (2.5 ng/ml) and DCPIB (20 µM). Scale bar: 500 µm. Wound closure was quantified using ImageJ software 24 h after wounding. Values are expressed as the ratio of the initial wound area and were obtained from six measurements (three independent experiments). Kruskal-Wallis with Dunn’s multiple comparison post hoc test was used with *p < 0.05. **e** Independent series of wound healing assay were performed in HK2 cells to illustrate the effect of LRRC8A silencing (siLRRC8A) in migration processes (six measurements from two independent experiments; Kruskal-Wallis with Dunn’s multiple comparison post hoc test with ***p < 0.001).

We also explored the cellular morphology changes induced by TGFβ1 treatment (24 h). Control cells, DCPIB-treated cells and siLRRC8A HK-2 cells exhibited a classical cuboidal epithelial shape (Fig. 6a). In contrast, cells that were treated with TGFβ1 (2.5 ng/ml, 24 h) changed to a spindle-shaped mesenchymal morphology. In siLRRC8A-transfected and DCPIB-treated cells, the TGFβ1-induced morphological changes were significantly less pronounced. Analysis of the circularity index (considering a value of 1 as a perfect circle and 0 as an infinitely elongated polygon) and aspect ratio (major axe divided by minor axe of the cell) confirmed the morphological changes: TGFβ1 induced a significant decrease in circularity and an increase in the aspect ratio (Fig. 6b). Targeting LRRC8/VRAC function either by using a pharmacological inhibitor (DCPIB) or by LRRC8-siRNA-mediated knockdown reversed the modifications of these parameters induced by TGFβ1 treatment.

Another characteristic of EMT is the development of the migratory and invasive ability of the cells. To assess cellular motility and migratory capacity, we performed a wound healing assay at 24 h (Fig. 6c, d). Upon treatment with TGFβ1, cells were able to close the wound better than control cells; nevertheless, cotreatment with DCPIB strongly inhibited the closure of the gap between the two cellular rows (Fig. 6c, d). Wound closure is also impaired in TGFβ1-treated cells transfected with siLRRC8A compared to those transfected with negative control siRNA (Fig. 6e).

Taken together, our results showed that LRRC8/VRAC channels, through their native permeability to GSH, regulate the cellular oxidative status and play a critical role in EMT.

## Discussion

Since the discovery of VRAC in the early 1980s, its features have impressed scientists and prompted them to examine what appeared as an enigma^25,26^. The finding of the molecular identity of VRAC as heteromers of LRRC8 proteins has finally solved this mystery^2,3^. Indeed, unlike most channels, LRRC8/VRAC has been reported to be permeable mainly to chloride ions but also to many anions and osmolytes^27^. Surprisingly, some transported osmolytes are uncharged, such as taurine, while some are positively charged, such as lysine^6^. VRAC is also permeable to negatively charged glutamate and gluconate, which are large molecules for channel permeation. Herein, we assessed the human LRRC8/VRAC permeability to the negatively charged tripeptide GSH, the main antioxidant agent within cells. In 2013, before the discovery of the VRAC molecular identity, Sabirov et al. showed that the osmosensitive release of GSH was significantly inhibited by blockers of VRAC in rat thymocytes^19^. The idea that VRAC is permeable to GSH is reinforced by the fact that i) connexin hemichannels (which have a similar structural organization with LRRC8) are permeable to GSH^15,28,29^ and ii) GSH exhibits a Stokes radius of 0.56 nm that is compatible with the passage through LRRC8/VRAC channels^5,30^.

In this study, we showed in the HEK293 cell line that GSH release occurred during hypotonic exposure, which was inhibited by the VRAC inhibitor DCPIB and was absent in LRRC8A-KO cells. More importantly, the time course of GSH release is perfectly consistent with the time course of RVD and chloride current activation we have previously described in the same condition^10^. Osmotic swelling-stimulated GSH release has already been reported in a perfused whole liver model with a time course roughly paralleling volume regulatory K^+^ efflux^18^. Assessment of LRRC8/VRAC GSH conductance using a patch-clamp approach demonstrated that cells submitted to hypotonic condition developed a current with VRAC biophysical features: outward rectification, inactivation during the stimulation at positive potentials and sensitivity to DCPIB as described previously^3,10^. The same observation was found in HK-2 epithelial cells which have 1.4 times more currents at +60 mV but exhibited a significatively higher cell capacitance (Fig. S2c) than HEK293 cells. VRACs in HK2 cells are permeable to GSH with a P_GSH_/P_Cl_ of 0.11 ± 0.02. This value is close to the one calculated for HEK293 cells (P_GSH_/P_Cl_ of 0.08 ± 0.01) and equivalent to value reported in rat thymocytes (P_GSH_/P_Cl_ of 0.10)^19^. The differences in relative permeability between the different cell types (Fig. S2b) could indicate that VRAC GSH permeability might present some variations depending on the cell type and the expression level of the different LRRC8 subunits. Expression level of LRRC8A subunit mRNA, was higher in HK2 cells compare to HEK293 cells. Taken together, our data showed that human LRRC8/VRAC is permeable to GSH and that GSH leaks outside the cell for a significant part from the LRRC8/VRAC during RVD.

In addition to hypotonic stress, VRAC can also be activated without cell swelling by other stimuli^8,23,24^. We found that TGFβ1 triggers a chloride current exhibiting biophysical characteristic of LRRC8/VRAC. The TGFβ1-induced VRAC current has a slower activation and a current amplitude at +100 mV weaker than the one recorded under hypotonic challenge. A mystery surrounding VRAC is what triggered the opening of the channel. Cell swelling leads to VRAC activation, but the exact mechanism remains completely unknown. ROS were proposed to activate VRAC^31–35^. Nevertheless, the hypothesis of VRAC activation by ROS does not fit with our results. First, HK2 cells mainly expressed LRRC8A/D subunits, and Gradogna et al. recently showed that these subunit combinations are more likely inhibited by ROS^36^. According to their work, only the assembly of LRRC8A/E is activated by ROS, as shown in the schematic view of their manuscript^10^. Second, a plethora of evidence in the literature indicates that VRAC activation is necessary for ROS production and that VRAC inhibition also blocks ROS accumulation within the cells^37–39^. The mechanism of TGFβ1-induced VRAC activation remains unknown, but our data suggest that it is prior to TGFβ1-induced ROS formation. In fact, our data showed that pharmacological inhibition of VRAC inhibited TGFβ1-induced GSH loss and impaired TGFβ1-induced ROS accumulation inside the cell. These results suggest that LRRC8/VRAC can modulate ROS levels inside the cell through its ability to permeate GSH. Indeed, GSH efflux has been widely reported to precede ROS production, and the leak of GSH under TGFβ1 might be the cause of the increase of ROS production^40^.

Targeting proteins involved in GSH transport to modulate ROS levels is of interest in many physiological and pathophysiological situations in which oxidative stress is involved. We have previously reported that CFTR activity through its permeability to GSH controls the cellular redox state and governs the destiny of the epithelial cells^34,41,42^. Connexin43 hemichannel-mediated loss of GSH has been shown to contribute to the oxidative stress and disassembly of cell junctions in renal tubular epithelial cells exposed to Ca^2+^-free medium^17^.

Oxidative stress is a crucial step in inducing EMT. EMT is characterized by the loss of epithelium integrity starting by the loss of cell-to-cell junction and a gain of mesenchymal features that leads to the disruption of the basal membrane, the stiffening of the extracellular matrix and the invasion of adjacent tissues. TGFβ1 is known to trigger EMT. In vitro experiments showed that TGFβ1 induced EMT of kidney tubular epithelial cells such as rat NRK52E^43^, mouse NP-1^44^ and human HK-2 cells^45,46^. In their rat model^21^, Rhyu et al. demonstrated that TGFβ1 induced EMT through the production of ROS and that the effect of TGFβ1 could be reproduced using H_2_O_2_. We confirmed the role of GSH and ROS levels in TGFβ1-treated HK2 cells using the GSH precursor NAC, which inhibited the TGFβ1-induced EMT response. We have also shown that DCPIB inhibits TGFβ1-induced GSH loss and ROS increase. Both pharmacological inhibition with DCPIB and LRRC8A gene silencing significantly inhibited TGFβ1-induced modulation of EMT-related marker expression, morphological changes, and increased cell migration. However, DCPIB alone affected ECAD, FN and COL4 mRNA expression suggesting off target effects of this molecule since those effects were not observed in HK2 cells exhibiting a functional inhibition of LRRC8A activity (siLRRC8A).

Our results showed that LRRC8A/VRAC may be a potential target to protect tubular cells from the loss of epithelial phenotype during EMT. In the context of cancer, pharmacological inhibition of VRAC has been reported to reduce cell viability, proliferation, migration, and invasion of human glioblastoma cell lines^47^. VRAC inhibition has also been described to protect cells from cerebral ischaemic damage^48–51^ and from hyperglycaemia-induced ROS production and apoptosis in cardiomyocytes^38,39^.

In conclusion, our data demonstrate the ability of LRRC8/VRAC to transport GSH, especially during EMT, and suggest that targeting VRAC may counteract the deleterious effect of oxidative stress in pathophysiological situations.

## Supplementary information

suppl. Figure 1

suppl. Figure 2

suppl. Figure 3

suppl. Figure 4

suppl. Figure 5
