# Supplementary figures and images for: LRRC8/VRAC channels exhibit a noncanonical permeability to glutathione, which modulates epithelial-to-mesenchymal transition (EMT)
Source: Cell Death Dis. 2019 Dec 5;10(12):925. doi: 10.1038/s41419-019-2167-z (PMC6895240; doi:10.1038/s41419-019-2167-z)

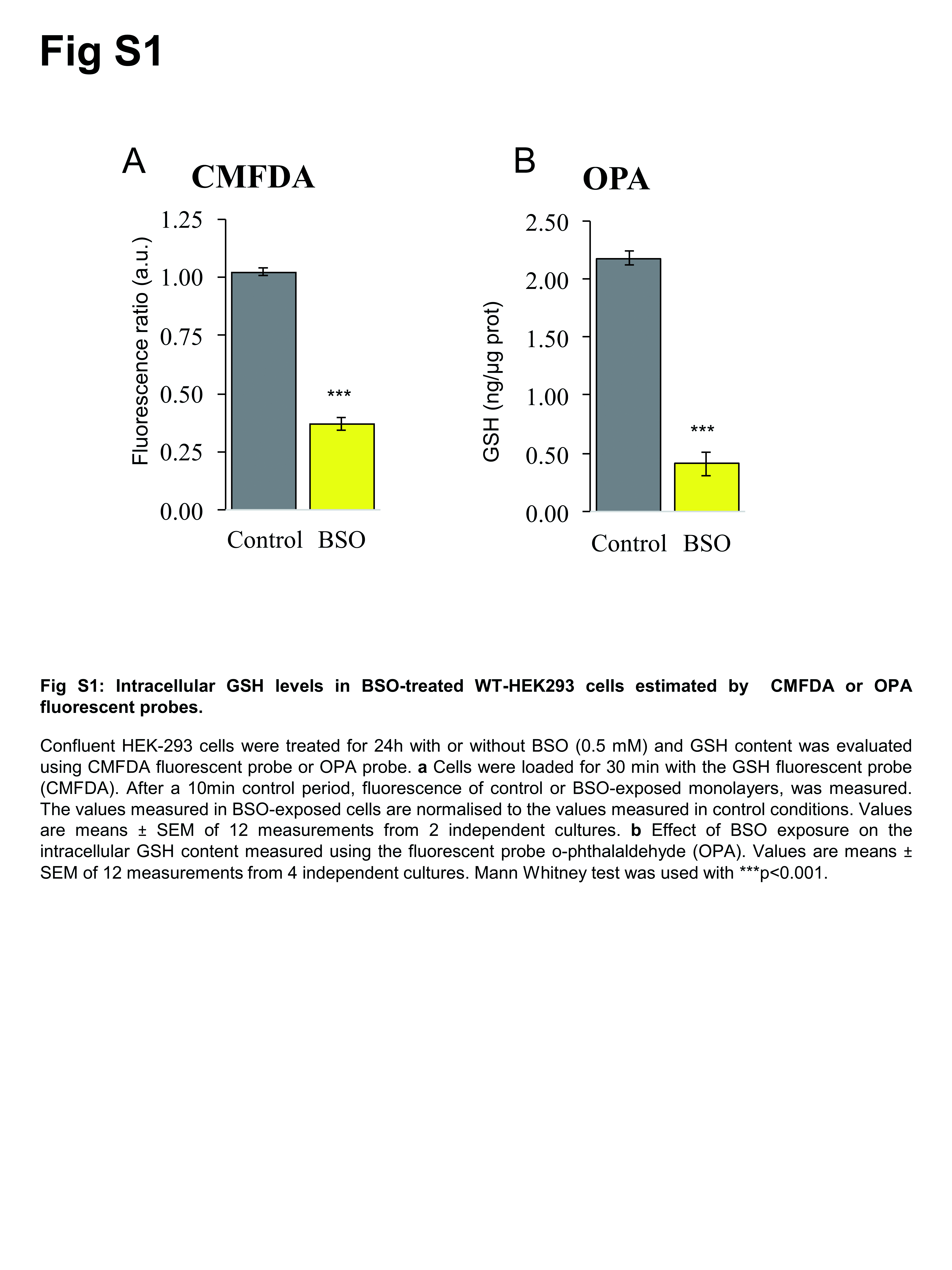

Supplement: Supplementary file 1 — suppl. Figure 1 [file 41419_2019_2167_MOESM1_ESM.tif]

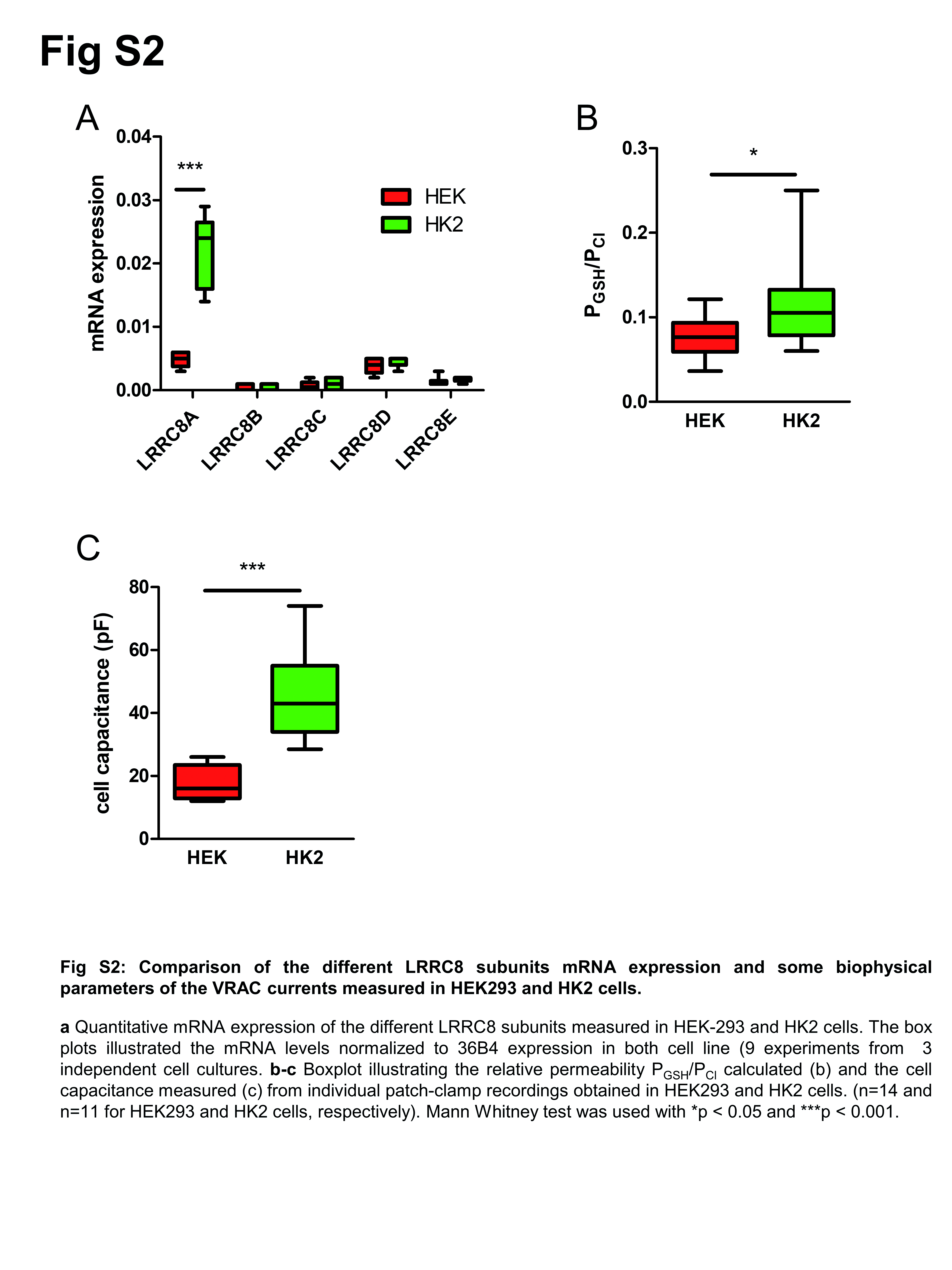

Supplement: Supplementary file 2 — suppl. Figure 2 [file 41419_2019_2167_MOESM2_ESM.tif]

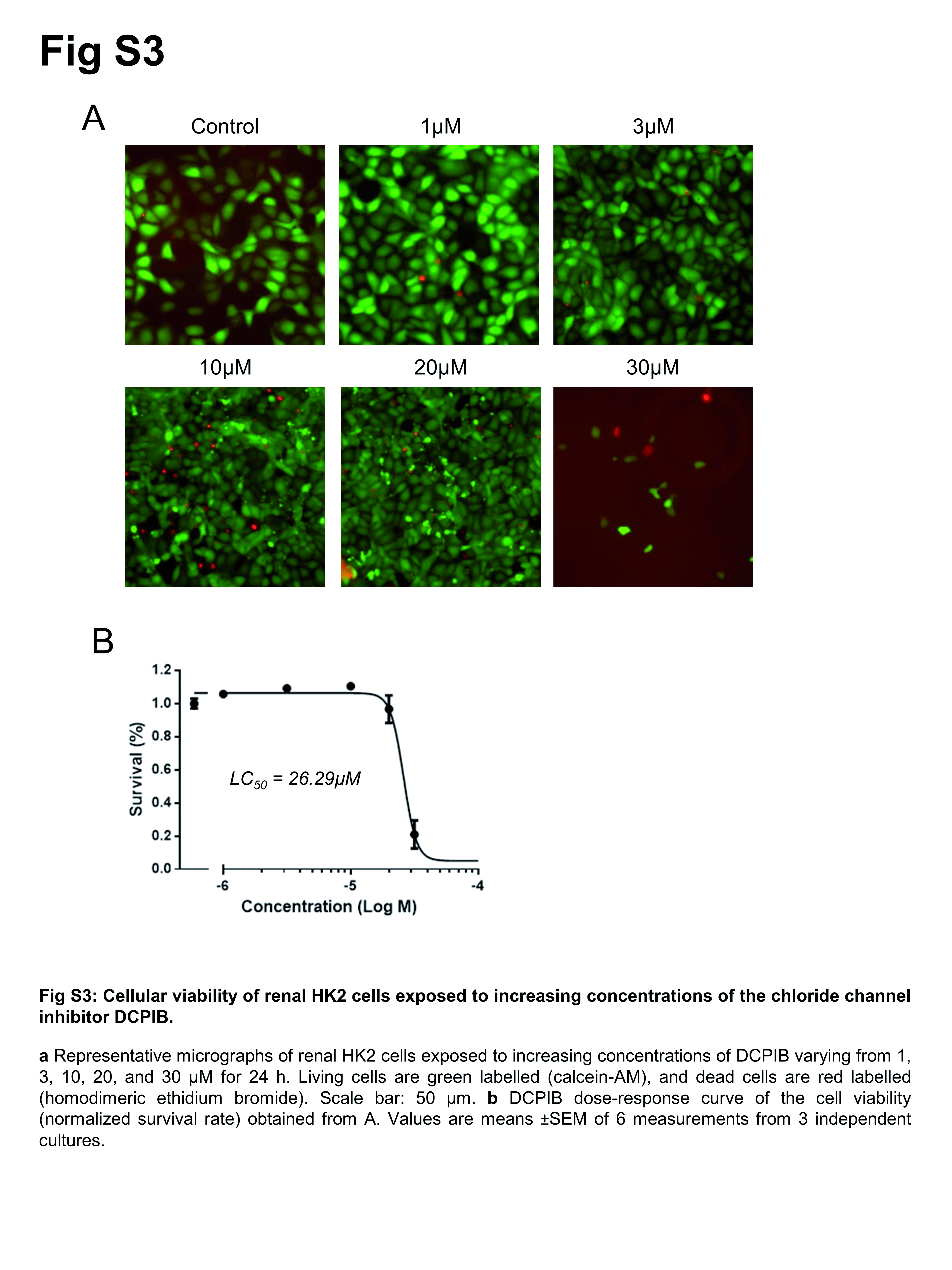

Supplement: Supplementary file 3 — suppl. Figure 3 [file 41419_2019_2167_MOESM3_ESM.tif]

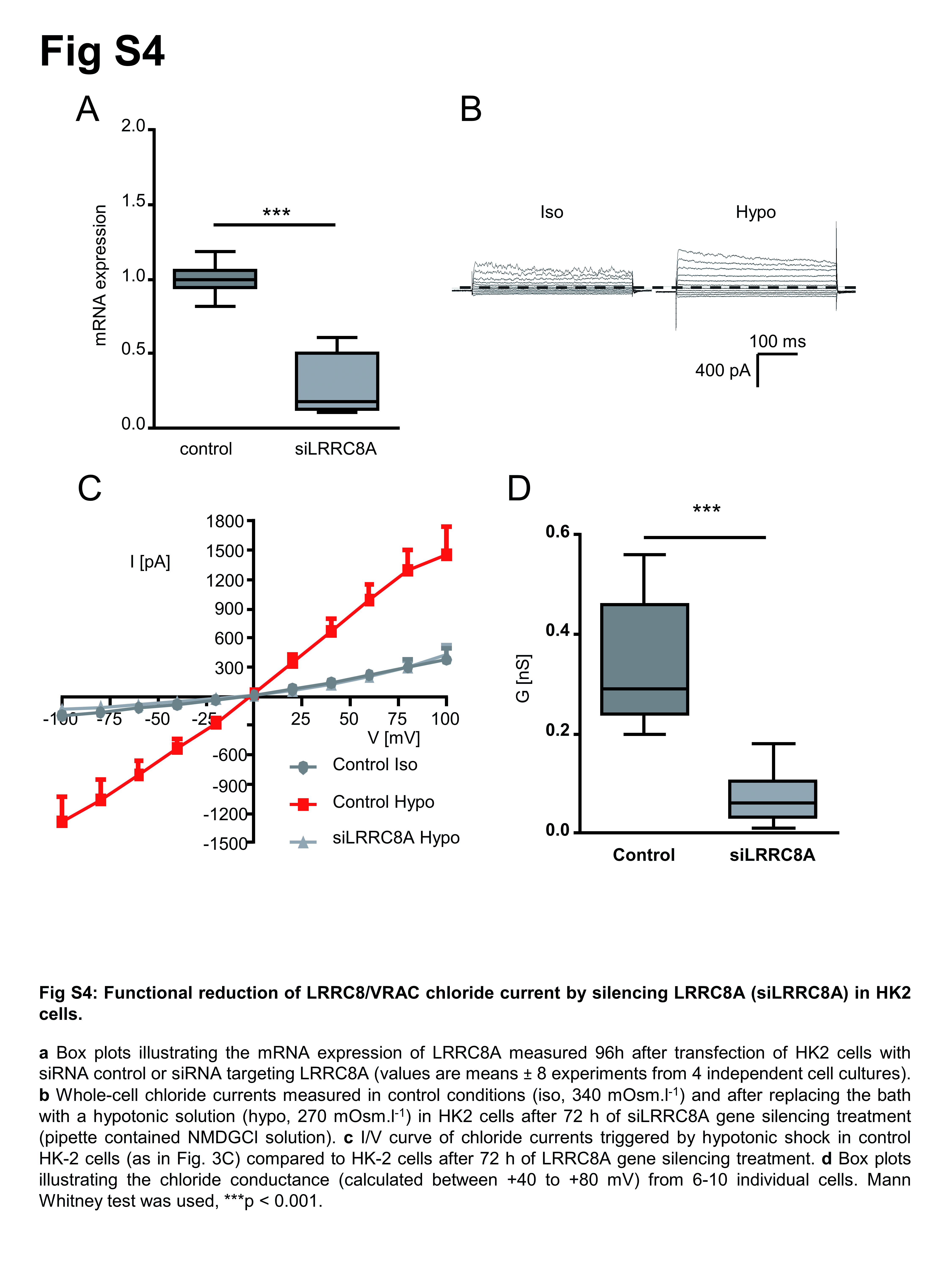

Supplement: Supplementary file 4 — suppl. Figure 4 [file 41419_2019_2167_MOESM4_ESM.tif]

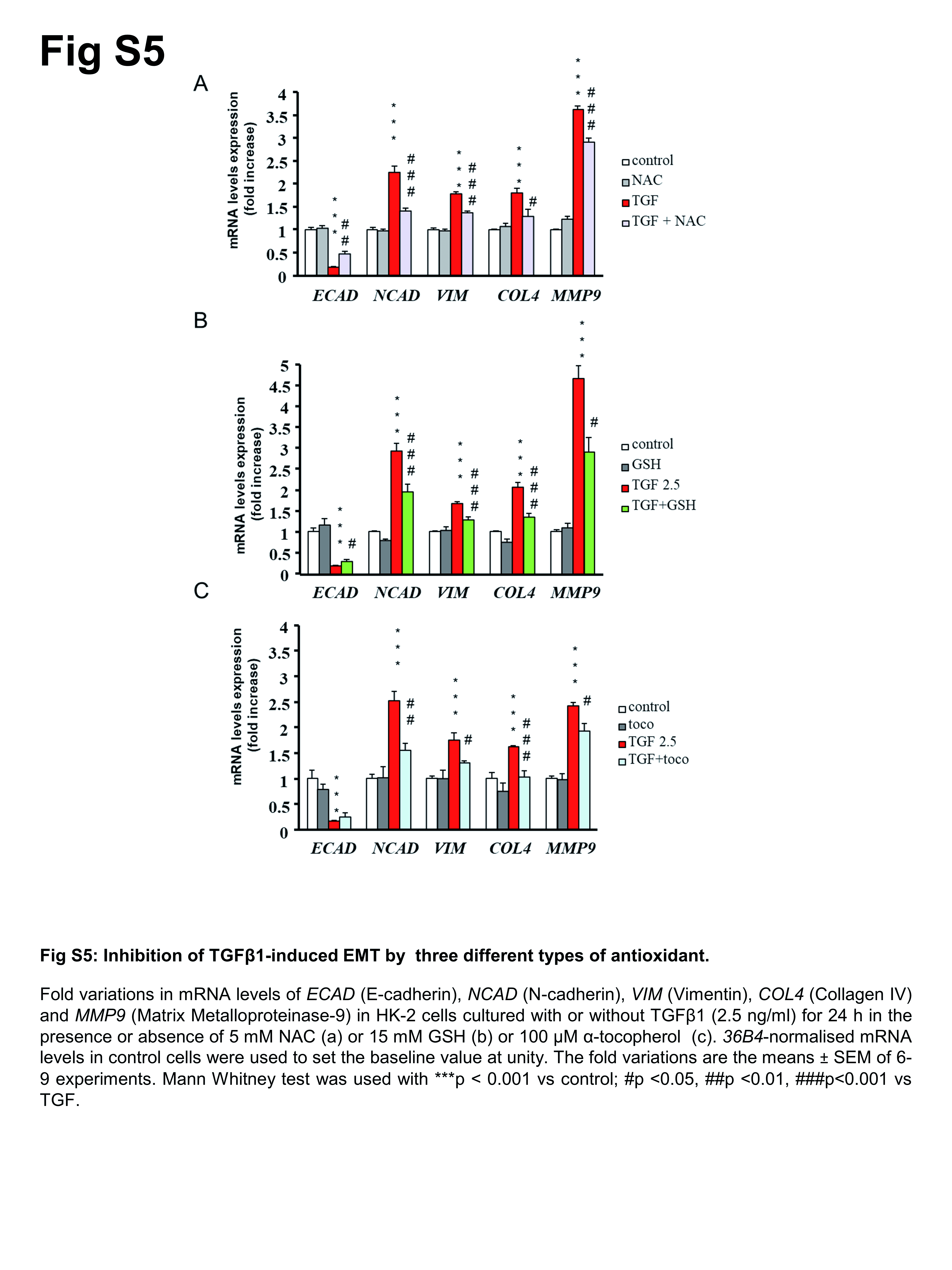

Supplement: Supplementary file 5 — suppl. Figure 5 [file 41419_2019_2167_MOESM5_ESM.tif]
